# Fatal hemorrhagic pneumonia in patients with hematologic diseases and Stenotrophomonas maltophilia bacteremia: a retrospective study

**DOI:** 10.1186/s12879-021-06420-0

**Published:** 2021-07-31

**Authors:** Lixia Zhu, Lulu Wang, Yuping Zhang, Rongrong Chen, Xueying Li, Jianai Sun, De Zhou, Mingyu Zhu, Xiaolong Zheng, Li Li, Jingjing Zhu, Mixue Xie, Xiudi Yang, Wenjuan Yu, Hongyan Tong, Honghu Zhu, Wanzhuo Xie, Jie Jin, Xiujin Ye

**Affiliations:** 1 Department of Hematology, The First Affiliated Hospital, College of Medicine, Zhejiang University, Hangzhou, 310003 Zhejiang Province China; 2 Department of Laboratory Medicine, The First Affiliated Hospital, College of Medicine, Zhejiang University, Hangzhou, 310003 Zhejiang Province China

**Keywords:** Stenotrophomonas maltophilia, Bacteremia, Hematologic diseases, Hemorrhagic pneumonia, Mortality

## Abstract

**Background:**

Fatal hemorrhagic pneumonia is one of the most severe manifestations of Stenotrophomonas maltophilia (SM) infections. Here, we aimed to investigate the clinical characteristics of SM bacteremia and to identify the risk factors of hemorrhagic pneumonia caused by SM in patients with hematologic diseases.

**Methods:**

The clinical records of 55 patients diagnosed with hematologic diseases and SM bacteremia were retrospectively reviewed. We compared patients’ clinical characteristics and outcomes between the hemorrhagic pneumonia group and non-hemorrhagic pneumonia group.

**Results:**

Twenty-seven (49.1%) patients developed hemorrhagic pneumonia. The overall mortality rate of SM bacteremia was 67.3%. Hemorrhagic pneumonia (adjusted HR 2.316, 95% CI 1.140–4.705; *P* = 0.020) was an independent risk factor of 30-day mortality in hematological patients with SM bacteremia. Compared with the non-hemorrhagic pneumonia group, patients in the hemorrhagic pneumonia group were older and showed clinical manifestations as higher proportions of isolated SM in sputum culture, neutropenia and elevated procalcitonin (PCT). Multivariate analysis showed that neutropenia, high levels of PCT, prior tigecycline therapy within 1 month were independent risk factors associated with hemorrhagic pneumonia.

**Conclusions:**

Neutropenia, high level of PCT and prior tigecycline therapy within 1 month were significant independent predictors of hemorrhagic pneumonia in hematologic patients with SM bacteremia. Due to no effective antibiotics to prevent hemorrhagic pneumonia, prophylaxis of SM infection and its progression to hemorrhagic pneumonia is particularly important.

## Introduction

Stenotrophomonas maltophilia (SM) is a non-fermentative gram-negative bacterium without highly virulent widely found in nature, and rarely causes infection in the normal immune population [1]. However, SM has been becoming a common cause of opportunistic infections in immunocompromised patients, especially in patients diagnosed with hematologic diseases, and can cause serious infections, such as bacteremia or hemorrhagic pneumonia [2–6].

Due to the intrinsic resistance of SM to many broad-spectrum antimicrobial agents, it is difficult to determine the appropriate antibiotic treatment before the SM is detected [2, 7]. Therefore, previous studies have shown a very poor prognosis in the cases of SM bacteremia, and the overall mortality was ranged from 35 to 75% [8–12]. Noteworthy, in patients with hematologic malignancies and/or hematopoietic stem cell transplantation (HSCT) recipients, SM can cause fatal hemorrhagic pneumonia, and the mortality is nearly 100% [10, 12–17]. At present, only a few reports have performed a statistical analysis to identify the risk factors of hemorrhagic pneumonia caused by SM [10, 12].

In this study, we identified cases of SM bacteremia in patients with hematologic disorders and performed a retrospective review of these patients’ clinical characteristics and outcomes. Further, we attempted to confirm the risk factors of SM bacteremia with hemorrhagic pneumonia.

## Materials and Methods

### Patients

This study was conducted at a 318-bed hematology department, the first affiliated hospital, College of Medicine, Zhejiang University, in China. From January 2015 to July 2019, all patients who were diagnosed with hematologic diseases and had positive blood cultures for SM were included in the study, with each patient being included only once at the time of initial blood culture. All patients were categorized into 2 groups: hemorrhagic pneumonia group and non-hemorrhagic pneumonia group. Clinical and microbiological characteristics were compared between the two groups to identify the risk factors for hemorrhagic pneumonia in SM bacteremia patients.

### Data collection

We retrospectively reviewed the electronic medical records for all enrolled patients and extracted the clinical data. The data included the following: age, gender, underlying hematologic disease, therapeutic treatment for hematologic diseases, sputum culture, polymicrobial bacteremia, laboratory tests, antibiotics susceptibility testing results, prior antibiotic therapy within 1 month, indwelling central venous catheter, inadequate initial empirical antimicrobial treatment and mortality.

### Definitions

SM bacteremia was defined as one or more positive blood cultures with clinical symptoms of infection. SM bacteremia with hemorrhagic pneumonia was diagnosed as follows:1) SM isolated from a blood culture, with or without SM in sputum culture at the same time, 2) newly detected imaging findings by chest X-ray or computed tomography (CT) scan when the blood culture was positive, 3) with pulmonary hemorrhage symptoms, such as continuous blood in sputum or hemoptysis. Polymicrobial bacteremia was defined as the isolation of another pathogen within 24 h of index SM isolate, satisfying the Centers for Disease Control and Prevention (CDC) criteria for blood stream infection (BSI) [18]. Neutropenia was defined as an absolute neutrophil count of < 500/uL at the onset of bacteremia. Adequate initial antimicrobial treatment was defined as the administration of at least one intravenous antibiotic to which the microorganism was susceptible in vitro within 72 h of the onset of SM bacteremia. Onset of SM bacteremia was defined as the date when the blood culture was collected.

### Blood culture and drug sensitivity test

The BacT/Alert 3D (bioMérieux, France) automated blood culture system was used. Species identification was performed by the automated Vitek 2 system with the matched gram-negative (GN) test card (bioMérieux, France). Antimicrobial susceptibility was tested with the agar dilution method. The antibiotics tested included trimethoprim-sulfamethoxazole (TMP-SMX), cefoperazone/sulbactam, minocycline, levofloxacin and tigecycline. The MICs of antimicrobial agents were interpreted according to the 2018 Clinical and Laboratory Standards Institute (CLSI) criteria except for tigecycline [19]. The interpretation of tigecycline was performed based on the breakpoints published by the Food and Drug Administration (FDA) (available at https://www.fda.gov/drugs/development-resources/tigecycline-injection-products), and the relevant criteria for *Enterobacteriaceae* was used. For *Enterobacteriaceae* isolates, ≤2 / ≥8 μg/ml were the sensitivity/resistance MIC breakpoints against tigecycline based on FDA criteria. Additionally, Cefoperazone/sulbactam susceptibility was based on the breakpoints for cefoperazone alone against *Enterobacteriaceae* (susceptible 16/8 mg/L; intermediate, 32/16 mg/L; and resistant, 64/32 mg/L) according to the criteria for susceptibility testing by CLSI [20].

### Statistical analysis

All statistical analyses were performed using SPSS version 22.0 for Windows. The independent sample t-test was used for continuous variables and the Chi-square test or Fisher’s exact test was used for categorical variables. Cox’s proportional hazard model with forward selection was used to find out which independent risk factors were predicting 30-day mortality. All variables with *P*-value of less than 0.10 in univariate analyses were enrolled in further multivariate analysis. The survival curves were performed by the Kaplan-Meier method. Univariate and multivariable logistic regression was used to evaluate the risk factors of hemorrhagic pneumonia in hematologic patients with SM bacteremia. Variables with *P*-value of less than 0.10 in univariate analyses were included in a multivariable logistic regression analysis. In addition, thrombocytopenia and prolonged activated partial thromboplastin time (APTT) which are considered as the potential variables associated with bleeding were also enrolled in the multivariate model. Results from the univariate and multivariate analysis are expressed as a hazard ratio (HR) or an odds ratio (OR) and 95% confidence interval (CI). Statistical tests were two-sided, and significance was defined as *P*-value < 0.05.

## Results

### Study population

Of the 288 patients with SM positive culture in the hematology department during the study period, 55 (19.1%) with SM bacteremia were included. The median age was 51 (range 12–72) years old and 56.4% were male in our study population. The clinical characteristics of this cohort were shown in Table 1. The most common underlying diseases diagnosed when admitted to hospital were acute myeloid leukemia (AML) (31 cases, 56.4%), followed by acute lymphoblastic leukemia (ALL) (12 cases, 21.8%), non-Hodgkin’s lymphoma (NHL) (6 cases, 10.6%), aplastic anemia (AA) (3 cases, 5.5%), hemophagocytic syndrome (HLH) (2 cases, 3.6%) and myelodysplastic syndrome (MDS)(1 case, 1.8%). Thirteen patients (23.6%) received HSCT. In addition to SM, 11 patients (20%) were also concomitant with other microbial bacteremia, included Klebsiella pneumonia in 3 cases, *Escherichia coli* in 3 cases, Staphylococcus haemolyticus in 2 cases, *Burkholderia cepacia* in 2 cases, *Staphylococcus epidermidis* in 1 case, and *Enterococcus faecium* in 1 case. Fifty-two (94.5%) patients with SM bacteremia had thrombocytopenia and 50 (90%) patients’ platelet counts were less than 50,000/uL. Bleeding was one of the most common clinical manifestations in this study. There were 30 (54.5%) patients with hemoptysis, 11 (20.0%) patients with gastrointestinal bleeding and 5 (9.1%) patients with intracranial hemorrhage. Hemorrhagic pneumonia was confirmed in 27(49.1%) patients. However, SM was only identified in 14 (25.5%) patients from sputum culture simultaneously.

**Table 1:** Demographic and clinical characteristics of hematologic patients with SM bacteremia

Characteristics	Patients (*N* = 55)	Hemorrhagic pneumonia (*n* = 27)	Non-hemorrhagic pneumonia (*n* = 28)	*P*-value^*^
Gender, male, No. (%)	31 (56.4)	17 (63.0)	14 (50.0)	0.333
Age, years, median (range)	51 (12–72)	56 (17–72)	48.5 (12–71)	0.049
Positive for SM in sputum culture, No. (%)	14 (25.5)	11 (40.7)	3 (10.7)	0.011
Polymicrobial bacteremia, No. (%)	11 (20.0)	4 (14.8)	7 (25.0)	0.345
Neutropenia, No. (%)	41 (74.5)	24 (88.9)	17 (60.7)	0.016
Hemoglobin < 60 g/L, No. (%)	31 (56.4)	16 (59.3)	15 (53.6)	0.671
Platelet < 50,000/uL, No. (%)	50 (90.9)	25 (92.6)	25 (89.3)	1.000
Fibrinogen< 2 g/L, No. (%)	11 (20.0)	7 (25.9)	4 (14.3)	0.281
PT > 13.5 s, No. (%)	22 (40.0)	14 (51.9)	8 (28.6)	0.078
APTT> 33.5 s, No. (%)	38 (69.1)	21 (77.8)	17 (60.7)	0.171
D-Dimer>700μg/L FEU, No. (%)	45 (81.8)	23 (85.2)	22 (78.6)	0.775
Albumin< 30 g/L, No. (%)	22 (40.0)	12 (44.4)	10 (35.7)	0.509
C-reactive protein≥10 mg/dl, No. (%)	31 (56.4)	17 (68.0)	14 (51.9)	0.236
Procalcitonin > 0.5μg/L, No. (%)	41 (74.5)	25 (92.6)	16 (57.1)	0.003
Hematological diseases				0.544
AML, No. (%)	31 (56.4)	15 (55.6)	16 (57.1)	
ALL, No. (%)	12 (21.8)	7 (25.9)	5 (17.9)	
NHL, No. (%)	6 (10.9)	3 (11.1)	3 (10.7)	
MDS, No. (%)	1 (1.8)	1 (3.7)	0 (0.0)	
AA, No. (%)	3 (5.5)	0 (0.0)	3 (10.7)	
HLH, No. (%)	2 (3.6)	1 (3.7)	1 (3.6)	
Chemotherapy in 30-day, No. (%)	43 (78.2)	21 (77.8)	22 (78.6)	0.943
Inadequate initial antimicrobial treatment, No. (%)	35 (63.6)	16 (59.3)	19 (67.9)	0.508
Central venous catheter, No. (%)	48 (87.3)	25 (92.6)	23 (82.1)	0.449
HSCT, No. (%)	13 (23.6)	6 (22.2)	7 (25.0)	0.808
Prior courses of chemotherapy, No. (%)				0.291
0	18 (32.7)	7 (25.9)	11 (39.3)	
≥ 1	37 (67.3)	20 (74.1)	17 (60.7)	
Chemotherapy in 30-day, No. (%)	43 (78.2)	21 (77.8)	22 (78.6)	0.943
30-day mortality, No. (%)	37 (67.3)	23 (85.2)	14 (50.0)	0.003

Abbreviations: *PT* prothrombin time, *APTT* activated partial thromboplastin time, *AML* acute myeloid leukemia, *ALL*, acute lymphoblastic leukemia, *NHL* non-Hodgkin’s lymphoma, *MDS* myelodysplastic syndrome, *AA* aplastic anemia, *HLH* hemophagocytic syndrome, *HSCT* hematopoietic stem cell transplantation

^*^Significant difference between hemorrhagic pneumonia group and non-hemorrhagic pneumonia group

The cohort was categorized into hemorrhagic pneumonia group and non-hemorrhagic pneumonia group (27 cases and 28 cases, respectively). The comparisons of clinical characteristics between the two groups were listed in Table 1. The median age was 56 years old in the hemorrhagic pneumonia group, which was older than the 48.5 years observed in the non-hemorrhagic pneumonia group (*P* = 0.049). There were more patients (40.7%) isolated SM from sputum in the hemorrhagic pneumonia group. The proportions of neutropenia and elevated procalcitonin (PCT) were significantly higher in the hemorrhagic pneumonia group than the non-hemorrhagic pneumonia group (*P* = 0.016 and *P* = 0.003, respectively). There were no significant differences for patients’ gender, polymicrobial bacteremia, anemia, platelet count, coagulation function, underlying hematologic diseases, C-reactive protein level, inadequate initial antimicrobial treatment and prior courses of chemotherapy between the two groups.

### Risk factors associated with mortality

The overall mortality was 67.3% (37/55), and all patients died within 30 days after SM bacteremia onset in non-survivor group. It was noteworthy that 20 (36.4%) patients died before obtained the positive blood culture reports, and all these patients had hemorrhagic pneumonia. The 30-day mortality rate was significantly higher in patients with hemorrhagic pneumonia than those without hemorrhagic pneumonia (85.2 and 50.0%, respectively). In this study, 35 (63.6%) patients received inadequate initial antimicrobial treatment. The 30-day mortality rate was 77.1% in patients who received inadequate initial antimicrobial treatment, which was significantly higher than that in patients treated with adequate initial antimicrobial (50.0%).

**Fig. 1:**
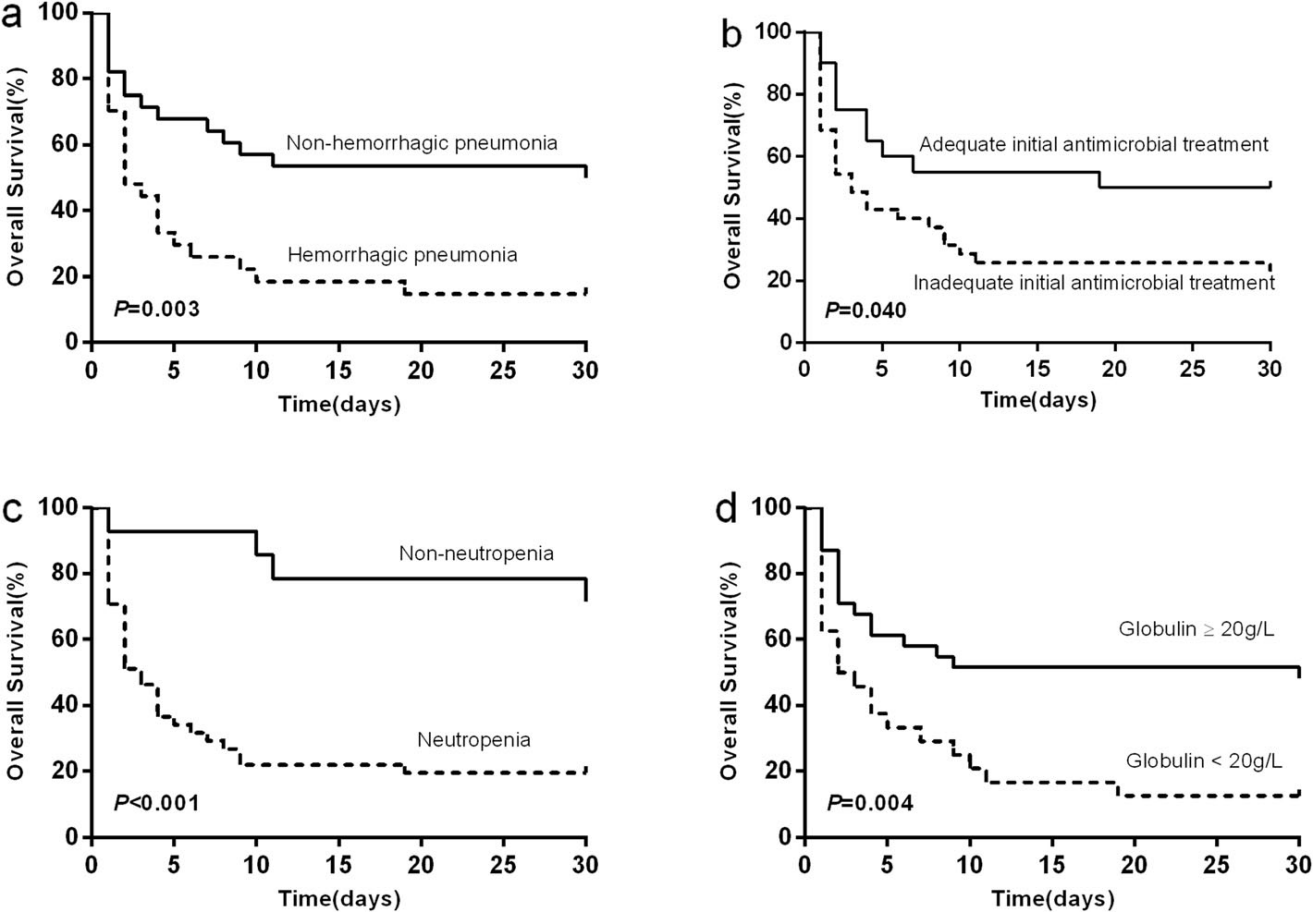
Kaplan-Meier curves of 30-day overall survival (OS) in patients with SM bacteremia. OS was significantly lower in patients with hemorrhagic pneumonia (**A**), inadequate initial antimicrobial treatment (**B**), neutropenia (**C**) and globulin less than 20 g/L (**D**)

The results of Cox’s proportional hazard analysis of risk factors associated with 30-day mortality in hematological patients with SM bacteremia was shown in Table 2. Neutropenia (adjusted HR 5.857, 95% CI 1.956–17.538; *P* = 0.002), globulin< 20 g/L (adjusted HR 2.931, 95% CI 1.478–5.811; *P* = 0.002), inadequate initial antimicrobial treatment (adjusted HR 2.974, 95% CI 1.368–6.465; *P* = 0.006) and hemorrhagic pneumonia (adjusted HR 2.316, 95% CI 1.140–4.705; *P* = 0.020) were the independent high-risk factors associated with 30-day mortality. Kaplan-Meier survival curves stratified by these four independent risk factors were shown in Fig. 1.

**Table 2:** Univariate and multivariate analysis of risk factors associated with 30-day mortality in hematological patients with SM bacteremia

Characteristics	Univariate analysis		Multivariate analysis	
	Hazard ratio (95% CI)	*P*-value	Hazard ratio (95% CI)	*P*-value
Age	1.014 (0.992–1.036)	0.205		
Polymicrobial bacteremia	1.265 (0.595–2.688)	0.541		
Neutropenia	5.100 (1.781–14.606)	0.002	5.857 (1.956–17.538)	0.002
Hemoglobin < 60 g/L	1.585 (0.812–3.092)	0.177		
Platelet< 50,000/uL	5.720 (0.781–41.863)	0.086		
Fibrinogen< 2 g/L	1.525 (0.718–3.241)	0.273		
PT > 13.5 s	2.575 (1.326–5.002)	0.005		
APTT> 33.5 s	1.694 (0.798–3.595)	0.170		
D-Dimer>700μg/L FEU	1.609 (0.626–4.133)	0.323		
Albumin< 30 g/L	1.336 (0.699–2.552)	0.381		
Globulin< 20 g/L	2.400 (1.241–4.641)	0.009	2.931 (1.478–5.811)	0.002
C-reactive protein≥10 mg/dl	1.397 (0.693–2.815)	0.349		
Procalcitonin > 0.5μg/L	1.827 (0.799–4.175)	0.153		
HSCT	1.385 (0.669–2.868)	0.381		
Inadequate initial antimicrobial treatment	2.014 (0.972–4.175)	0.060	2.974 (1.368–6.465)	0.006
Central venous catheter	1.183 (0.460–3.041)	0.728		
Prior tigecycline therapy within 1 month	1.043 (0.544–2.001)	0.898		
Prior carbapenem therapy within 1 month	0.946 (0.394–2.271)	0.902		
Hemorrhagic pneumonia	2.496 (1.268–4.914)	0.008	2.316 (1.140–4.705)	0.020

Abbreviations: CI, confidence interval; PT, prothrombin time; APTT, activated partial thromboplastin time; HSCT, hematopoietic stem cell transplantation

### Previous antimicrobial therapy within 1 month before BSI onset

All patients in this study were administrated with antibiotics before SM bacteremia onset, either due to a previous other infection or as an empirical therapy for unknown origin infection (Table 3). The majority of patients received carbapenems (46 cases, 83.6%) and/or antifungal drugs (45 cases, 81.8%). But there were only a few patients who had treated with TMP-SMX (3 cases, 5.5%). It was to be observed that a total of 23 (41.8%) cases received tigecycline, and tigecycline was more frequently prescribed in the hemorrhagic pneumonia group (59.3 and 25%, respectively, *P* = 0.010).

**Table 3:** Previous antimicrobial therapy within 1 month before SM bacteremia onset

Antibiotics	Total (*N* = 55)	Hemorrhagic pneumonia(*N* = 27)	Non-hemorrhagic pneumonia (*N* = 28)	*P*-value
Carbapenems, No. (%)	46 (83.6)	23 (85.2)	23 (82.1)	1.000
^a^Anti-MRSAs, No. (%)	38 (69.1)	16 (59.3)	22 (78.6)	0.121
Aminoglycoside, No. (%)	5 (9.1)	3 (11.1)	2 (7.1)	0.966
Tigecycline, No. (%)	23 (41.8)	16 (59.3)	7 (25.0)	0.010
Fluoroquinolones, No. (%)	11 (20.0)	6 (22.2)	5 (17.9)	0.686
TMP-SMX, No. (%)	3 (5.5)	0 (0.0)	3 (10.7)	0.236
Piperacillin/tazobactam, No. (%)	15 (27.3)	10 (37.0)	5 (17.9)	0.110
Cefoperazone/sulbactam, No. (%)	30 (54.5)	14 (51.9)	16 (57.1)	0.694
^b^Anti-fungles, No. (%)	45 (81.8)	20 (74.1)	25 (89.3)	0.266

^a^Anti-MRSAs included glycopeptides, linezolid, and daptomycin

^b^Anti-fungles included fluconazole, voriconazole, posaconazole, caspofungin and micafungin

### In vitro antimicrobial susceptibility

**Fig. 2:**
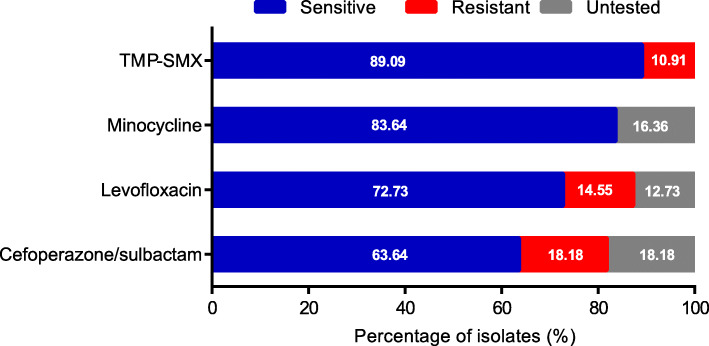
In vitro antimicrobial susceptibility for SM isolates

The antimicrobial susceptibilities of SM isolate in vitro are shown in Fig. 2. All patients were tested for TMP-SMX sensitivity, with a sensitivity rate of 89.09%. Susceptibility to levofloxacin, minocycline, cefoperazone/sulbactam was 83.3% (40/48), 100% (46/46) and 77.8% (35/45), respectively. There was no significant difference in susceptibility to four antibiotics mentioned above between the hemorrhagic pneumonia group and the non-hemorrhagic pneumonia group. In the present study, only six cases performed susceptibility tests to tigecycline, and the susceptibility is 83.3% (5/6).

### Risk factors associated with hemorrhagic pneumonia

For patients with SM bacteremia, once hemorrhagic pneumonia develops, the patients’ conditions always rapidly deteriorate and nearly all these patients die in a very short time. Univariate analysis showed that neutropenia, high levels of PCT (> 0.5μg/L) and prior tigecycline therapy within 1 month were significant risk factors for hemorrhagic pneumonia in SM bacteremia patients. Variables finally included in the multivariate analysis were neutropenia, platelet< 50,000/uL, prothrombin time (PT) > 13.5 s, APTT> 33.5 s, high levels of PCT (> 0.5μg/L) and prior tigecycline therapy within 1 month. In multivariate analysis showed in Table 4, independent risk factors associated with hemorrhagic pneumonia were neutropenia (OR = 4.988, 95% CI 1.040–23.920; *P* = 0.045), high levels of PCT (OR = 11.322, 95% CI 1.943–65.956; *P* = 0.007), prior tigecycline therapy within 1 month (OR = 4.482, 95% CI 1.135–17.702; *P* = 0.032).

**Table 4:** Univariate and multivariate analysis of risk factors associated with hemorrhagic pneumonia in hematological patients with SM bacteremia

Characteristics	Univariate analysis		Multivariate analysis	
	OR (95% CI)	*P*-value	OR (95% CI)	*P*-value
Age	1.030 (0.994–1.068)	0.105		
Polymicrobial bacteremia	1.917 (0.490–7.494)	0.350		
Neutropenia	5.176 (1.252–21.411)	0.023	4.988 (1.040–23.920)	0.045
Hemoglobin < 60 g/L	1.261 (0.433–3.668)	0.671		
Platelet< 50,000/uL	1.500 (0.230–9.763)	0.671		
Fibrinogen< 2 g/L	2.100 (0.537–8.217)	0.286		
PT > 13.5 s	2.692 (0.883–8.206)	0.082		
APTT> 33.5 s	2.265 (0.694–7.389)	0.175		
D-Dimer>700μg/L FEU	1.568 (0.389–6.319)	0.527		
Albumin< 30 g/L	1.440 (0.487–4.255)	0.509		
Globulin< 20 g/L	1.261 (0.433–3.668)	0.671		
C-reactive protein≥10 mg/dl	1.973 (0.638–6.106)	0.238		
Procalcitonin > 0.5μg/L	9.375 (1.849–47.522)	0.007	11.322 (1.943–65.956)	0.007
HSCT	1.167 (0.335–4.060)	0.809		
Inadequate initial antimicrobial treatment	1.451 (0.481–4.377)	0.508		
Central venous catheter	2.717 (0.479–15.402)	0.259		
Prior tigecycline therapy within 1 month	4.364 (1.383–13.772)	0.012	4.482 (1.135–17.702)	0.032
Prior carbapenem therapy within 1 month	1.250 (0.297–5.256)	0.761		

Abbreviations: *OR* odds rate, *CI* confidence interval, *PT* prothrombin time; *APTT* activated partial thromboplastin time, *HSCT* hematopoietic stem cell transplantation

## Discussion

In the present study, we found two main results. First, the mortality rate of SM bacteremia with hemorrhagic pneumonia was significantly higher than those without hemorrhagic pneumonia. Hemorrhagic pneumonia was an independent risk factor of 30-day mortality in hematologic patients with SM bacteremia. Second, neutropenia, high level of PCT and prior tigecycline therapy were found to be independent risk factors associated with hemorrhagic pneumonia in hematologic patients with SM bacteremia.

Hemorrhagic pneumonia is one of the most severe manifestations of SM infections. Almost all patients die within a few days after the onset of hemoptysis and before the isolate is detected from blood or sputum culture. Previous literature has proven that inadequate initial antimicrobial treatment is an independent adverse prognostic factor for SM bacteremia [21], which was confirmed again in our study. Nevertheless, the rate of receiving the adequate initial antimicrobial treatment was similar between hemorrhagic pneumonia group and non-hemorrhagic pneumonia group. This indicates that hemorrhagic pneumonia caused by SM is a severe condition and difficult to save a patient once it develops despite adequate initial antimicrobial treatment. In our study, there were 4 patients with successful treatment outcomes after early combination therapy with TMP/SMX, cefoperazone/sulbactam, moxifloxacin, and/or tigecycline, and rapid recovery of neutropenia. There are only two patients who were diagnosed with hematologic malignancies and hemorrhagic pneumonia due to SM infection and successfully treated after early combined therapy with TMP/SMX, polymyxin, and/or moxifloxacin were reported in the previous study [22]. Both patients had neutropenia during infection, and neutropenia resolved within 1 week after SM bacteremia onset. Rapid recovery of neutropenia depending on hematologic disorder itself and supportive treatment such as granulocyte colony stimulating factor (G-CSF), might be a key factor in reducing mortality of hemorrhagic pneumonia. However, there is still no effective therapy that could prevent life-threatening hemorrhagic pneumonia. The prophylaxis of SM infection and its progression to hemorrhagic pneumonia is thus crucially important. It is necessary to clarify risk factors for hemorrhagic pneumonia.

A previous review summarized the clinical information of 30 cases of hemorrhagic pneumonia caused by SM [10]. The authors found that severe neutropenia (< 100 uL) was the most important risk factor for hemorrhagic pneumonia induced by SM. In the present study as well, 24 of the 27(88.9%) SM bacteremia patients with hemorrhagic pneumonia were neutropenic (< 500 uL). This maybe explain why SM bacteremia is likely to occur in immunocompromised patients, and why hemorrhagic pneumonia induced by SM is always developed in patients with hematologic malignancy. Neutropenia due to the hematologic disorder itself or treatment for the hematologic disorder may be an important factor for susceptibility to SM bacteremia and progression to hemorrhagic pneumonia.

PCT is a prohormone of calcitonin, which has a short half-life and is highly upregulated during the acute phase of sepsis. PCT is valuable and sensitive in predicting bacteremia and distinguishing Gram-negative sepsis in hematologic patients with febrile neutropenia [23–27]. Its concentration is markedly associated with the severity of infection [28]. The biological action of PCT during severe infection is largely unknown. PCT might be a mediator of inflammation, which can be increased by overproduction of early proinflammatory cytokines, such as interleukin (IL)-1β, tumor necrosis factor (TNF)-α and IL-6. The high level of PCT indicated severity of infection, and might be associated with excessive inflammation which can cause vasculitic damage [29]. This maybe explain why patients with elevated PCT was more likely to progress and develop hemorrhagic pneumonia.

Tigecycline is a potential alternative antimicrobial for SM infection, and the susceptibility to tigecycline is ranged from 70.6 to 90.6% according to previous studies [30, 31, 32]. However, our study showed that prior tigecycline therapy was an independent risk factor for developing hemorrhagic pneumonia caused by SM, and empirical antimicrobial treatment with tigecycline cannot reduce the mortality rate (63.6%). The reason may be due to the side effects of tigecycline. It was reported by Windhorst et al. that StmPr1, a protease of the subtilase family secreted by SM, can degrade the collagen and fibroblasts in vitro*.* This process might lead to tissue invasion, destruction of the alveolar micro vessels and hemorrhage [33]. It was previously reported that the use of tigecycline might induce coagulopathy usually manifested as prolongation of PT and APTT and a reduction in the fibrinogen level in a dose-dependent manner [34]. The most severe adverse event is bleeding. This view was confirmed by our study again, which showed a higher incidence of prolonged APTT in patients received prior tigecycline therapy (87.0% vs 56.3%, *P* = 0.015, χ^2^ = 5.908). Furthermore, the majority of enrolled patients occurred thrombocytopenia concurrently in our study. If destruction of alveolar micro vessels by protease secreted by SM, coagulative dysfunction induced by tigecycline, and thrombocytopenia happen at the same time, the probability of hemorrhagic pneumonia occurring might increase. On the other hand, it was identified that the resistance to tigecycline was easily selected during exposure to this antimicrobial and cross-resistance to other antimicrobials, such as aztreonam and quinolones, was also presented [35]. Unfortunately, in our study, there were only six cases finished susceptibility test to tigecycline in vitro, and only one strain showed resistance. The rate of resistance to TMP-SMX was higher in the prior tigecycline therapy group (17.4% vs 6.3%), which was no significantly statistical difference (*P* = 0.223). However, there may be some bias based on the nature of retrospective study. Tigecycline is a broad-spectrum antibiotic used to treat infections that do not respond to first-line treatments. It might be indicated that these patients with prior tigecycline use had suffered from severe infections in a short time before SM bacteremia onset. It was unknown whether this previous infection was associated with hemorrhagic pneumonia. We need to verify the potential association between hemorrhagic pneumonia and prior tigecycline use by expanding the case samples or in a SM-infected mouse model.

The study still has several limitations. First of all, 59.3% of cases did not obtain a positive result of SM in sputum culture in the hemorrhagic pneumonia group. It might lead to the argument about whether SM was the true pathogen of hemorrhagic pneumonia. For this special group of patients, sputum culture was difficult to carry out under certain conditions, such as the sudden appearance of massive hemoptysis. Besides, the majority of these patients had thrombocytopenia, so it was unable to perform bronchoalveolar lavage fluid culture to improve the positive rate of culture. According to our definition, the blood cultures of all patients were positive for SM. Then combined with clinical and imaging findings, we reasonably identified that hemorrhagic pneumonia was caused by SM. On the other hand, the antimicrobial susceptibilities of SM isolates were insufficient. There were only a few cases completed the susceptibility test to tigecycline. Consequently, it is difficult to confirm the relationship between the susceptibility to tigecycline and the development of hemorrhagic pneumonia.

## Conclusions

Our study suggested that SM bacteremia with hemorrhagic pneumonia developed in patients with hematologic diseases was a condition with rapid progression and high mortality. In addition, neutropenia, high level of PCT and prior tigecycline therapy within 1 month were independent predictors of hemorrhagic pneumonia in hematologic patients with SM bacteremia, and thus patients with these conditions should be treated carefully. Futher studies are needed to explore effective approaches for prevention of the life-threatening hemorrhagic pneumonia in these patients.

## Data Availability

The datasets used during the current study will be available from the corresponding author on reasonable request and accepted by the Ethics Review Committee.

## Abbreviations

SM: Stenotrophomonas maltophilia
HSCT: hematopoietic stem cell transplantation
CT: computed tomography
CDC: Centers for Disease Control and Prevention
BSI: blood stream infection
GN: gram-negative
TMP-SMX: trimethoprim-sulfamethoxazole
CLSI: Clinical and Laboratory Standards Institute
FDA: Food and Drug Administration
APTT: activated partial thromboplastin time
HR: hazard ratio
OR: odds ratio
CI: confidence interval
AML: acute myeloid leukemia
ALL: acute lymphoblastic leukemia
NHL: non-Hodgkin’s lymphoma
AA: aplastic anemia
HLH: hemophagocytic syndrome
MDS: myelodysplastic syndrome
PCT: procalcitonin
OS: overall survival
PT: prothrombin time
G-CSF: granulocyte colony stimulating factor
IL: interleukin
TNF: tumor necrosis factor
